# A Protocol for Identifying Priorities for Women+ Health in the Maritime Provinces Using a Priority Setting Partnership Approach

**DOI:** 10.3390/healthcare14101287

**Published:** 2026-05-09

**Authors:** Justine Dol, Christine Pritchett, LeeAnn Larocque, James Bentley, Melissa Brooks, Annette J. Elliott Rose, Natalie O. Rosen, Emma Davies, Madhuri Yeluri, Meghan Gosse

**Affiliations:** 1 Centre for Pediatric Pain Research, IWK Health, Halifax, NS B3K 6R8, Canada; meghan.gosse@iwk.nshealth.ca; 2 Women and Newborn Health Program, IWK Health, Halifax, NS B3K 6R8, Canada; christine.pritchett@iwk.nshealth.ca (C.P.); jim.bentley@iwk.nshealth.ca (J.B.); melissa.brooks@iwk.nshealth.ca (M.B.); 3 Clinical Care, IWK Health, Halifax, NS B3K 6R8, Canada; leeann.larocque@iwk.nshealth.ca; 4 Department of Obstetrics and Gynaecology, Faculty of Medicine, Dalhousie University, Halifax, NS B3K 6R8, Canada; nrosen@dal.ca; 5 Clinical Care, Nova Scotia Health, Halifax, NS B3S 0H6, Canada; annettej.elliottrose@nshealth.ca; 6 Department of Psychology and Neuroscience, Faculty of Science, Dalhousie University, Halifax, NS B3H 4R2, Canada; 7 Independent Researcher, Halifax, NS B3K 6R8, Canada

**Keywords:** women+ health, research priority setting, patient-oriented research, James Lind Alliance, health equity, mixed-methods research, Maritime provinces, interest holder engagement

## Abstract

Background/Objectives: Women+ (e.g., women and individuals assigned female at birth) experience disproportionate health risks and persistent gaps in access to care. Women+ health research remains significantly underfunded and understudied, contributing to inequities in diagnosis, treatment, and outcomes. This study aims to collaboratively identify and prioritize the most pressing unanswered research questions related to women+ health in the Maritime provinces of Canada. Methods: This study will use a modified Priority Setting Partnership (PSP) methodology based on the James Lind Alliance framework. A mixed-methods participatory approach will be used, including bilingual online surveys (French, English) and a one-day consensus workshop. Participants will include women+, healthcare professionals, researchers, policymakers, and the public residing in the Maritime provinces (Nova Scotia, New Brunswick, and Prince Edward Island). An initial survey will collect research uncertainties through open-ended questions. A second survey will rank verified uncertainties, followed by a facilitated workshop to achieve consensus on the Top 10 research priorities. Descriptive statistics will summarize participant demographics. Anticipated Results: This project is expected to generate a collaboratively developed Top 10 list of research priorities for women+ health in the Maritimes, which will be used to prioritize future research related to women+ health. Conclusions: By centering women+ voices and engaging diverse interest holders, this study will establish a shared regional research agenda to guide future research, funding, and policy initiatives for women+ health research.

## 1. Introduction

The Maritime provinces of Nova Scotia, New Brunswick, and Prince Edward Island comprise 5.1% of the Canadian population, of which 50.7% identify as women+ [1]. Despite the relatively low population density, women+ in the Maritimes face disproportionate health risks compared to other Canadians, including having the highest incidence of breast and lung cancers [2,3]. Across Canada and the Atlantic provinces, the leading causes of death for women+ between 2019 and 2023 were cancer, cardiovascular disease, and cerebrovascular disease [4,5]. Addressing these disparities requires a clear understanding of regional women+ health priorities.

In this study, we use the term “women+” to reflect a gender-inclusive and additive approach to women’s health research. This framing acknowledges that not all individuals who experience health conditions traditionally associated with ‘women’ identify as a woman, while also maintaining the visibility of women as a distinct population in health research. Our approach aligns with recommendations by Brotto and Galea, which emphasize balancing inclusivity with the importance of not reducing individuals to biological characteristics alone [6]. The term women+ encompasses cisgender women as well as transgender individuals, non-binary, Two-Spirit, and others with diverse gender identities who may experience health concerns traditionally associated with women’s health or linked to sex-based biological characteristics across the lifespan.

Despite the critical importance, women+ health remains understudied and underfunded. In Canada, only 7% of national health research funding is allocated to women+ health [7]. Moreover, even when women+ are included in research, sex and gender are not consistently reported, contributing to ongoing gaps in knowledge and inequities in care [8]. For example, funding for research on non-reproductive organs (e.g., brain, heart) is six to seven times higher than for reproductive organs (e.g., breast, uterus) [9]. These gaps contribute to inequities in diagnosis, treatment, and health outcomes [10].

Healthcare delivery in the Maritime provinces involves both local provincial systems, including Nova Scotia Health, Horizon/Vitalité (New Brunswick), and Health PEI as well as IWK Health, which provides specialized and tertiary care to women and children across the Maritimes. Despite this infrastructure, access to primary care remains a persistent challenge. Recent data indicate that a substantial number of residents across the Maritime provinces are without a regular primary care provider, highlighting ongoing access gaps in the region [11,12,13]. National estimates further suggest that 11% of the population report unmet healthcare needs [14], with women+ reporting higher rates of unmet needs than men [15]. Emerging evidence suggests that gender-diverse individuals may face additional barriers in accessing appropriate care [16]. These gaps underscore the need for research that identifies the specific needs of women+ in this region.

In 2025, the IWK Foundation, a not-for-profit organization that raises funds to support IWK Health, a tertiary women’s and children’s health center in Atlantic Canada, conducted a region-wide consultation survey gathering over 27,000 responses from women+ across the Maritimes [17]. The consultation survey identified several commonly reported concerns, including systemic barriers to accessing primary and specialist care, long wait times, gaps in reproductive and menopause-related services, and challenges navigating the healthcare system [17]. While the survey provided valuable insights into community experiences and perceived gaps in care, it was not designed to systematically identify and prioritize researchable questions across interest holder groups. Building on this work, the present study describes the protocol and methodological approach to identify and prioritize the top research questions for women+ health in the Maritimes. Using a systematic, evidence-informed priority setting approach, the process will reflect the perspectives of women+, caregivers of women+, researchers, healthcare professionals, policy makers, and members of the public, centering women+ voices to guide future research, funding, and policy decisions and to strengthen regional collaboration for women+ health.

## 2. Materials and Methods

### 2.1. Study Design

**Figure 1:**
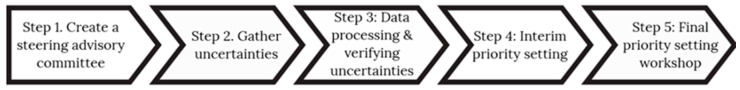
Overview of the Priority Setting Partnership (PSP) process, including the five key phases: (1) formation of the steering advisory committee; (2) collection of research uncertainties through an initial survey; (3) data processing and verification of uncertainties; (4) interim prioritization through a second survey; and (5) final consensus-building workshop to identify the Top 10 research priorities.

This study describes a protocol for identifying and prioritizing research questions related to women+ health in the Maritime provinces of Canada using a modified Priority Setting Partnership (PSP) approach informed by the James Lind Alliance (JLA) methodology [18,19]. The PSP approach is designed to bring together individuals with lived experience, caregivers, healthcare professionals, policymakers, and other interest holders to collaboratively identify and prioritize unanswered research questions of shared importance. The process is outlined in Figure 1 below.

While the core principles of the JLA methodology are maintained, adaptations are intended to enhance contextual relevance and inclusivity within the Maritime provinces, supporting the development of regionally meaningful research priorities and to enhance the feasibility of conducting the PSP within available time and resource constraints.

First, we did not exclude researchers from participating but rather asked them to self-report their identities. It is important to recognize that people hold multiple identities, with researchers, policy makers and health professionals also potentially identifying as a woman+. Nevertheless, there is potential bias in the process with the inclusion of researchers as participants which may influence the uncertainties identified and prioritization process, as researchers may prioritize questions which may differ from the priorities of individuals with lived experience. However, since each uncertainty is given the same weight during the initial development of uncertainties, combined with the single vote per participant in the ranking process, this risk is minimal. The prioritization process will be structured and facilitated to ensure that the perspectives of individuals with lived experience remain central and are not overshadowed by professional viewpoints.

Secondly, we are proposing the use of a hybrid (in-person and virtual) consensus workshop format to enhance accessibility across geographically dispersed provinces. This approach has been used in other PSPs including with cystic fibrosis [20], spinal cord injuries [21], and women’s health and technology [22].

### 2.2. Setting and Scope

The study will be conducted across the Canadian Maritime provinces of Nova Scotia, New Brunswick, and Prince Edward Island. Participants must reside in one of these provinces at the time of participation. While healthcare delivery models may differ across provincial systems, the PSP is designed to identify shared and regionally relevant research priorities in women+ health across the Maritimes.

The scope of the PSP focuses on women+ health across the adult life course and encompasses a broad range of physical, mental, emotional, social, and cultural health considerations. Areas of interest include, but are not limited to, reproductive and sexual health, chronic disease, mental health, perinatal and menopausal health, access to care, health system navigation, social determinants of health, and experiences within healthcare settings. The PSP will focus on identifying researchable uncertainties rather than addressing individual clinical concerns or providing medical advice.

To promote awareness of this study, a website (www.maritimewomenshealth.com) provides information on the project and updates as the study progresses.

### 2.3. Data Collection Overview

The PSP consists of five sequential phases. Ethical approval from IWK Health Research Ethics Board has been obtained (REB#1032488).

*Step 1: Create a steering advisory committee*

This PSP will be overseen by a Steering Group comprising women+, healthcare professionals, researchers, policy makers, and key interest holders in women+ health research in the Maritime Provinces. The steering committee is being led by first author (JD) and supported by a research coordinator (MG). The Steering Group’s responsibilities include refining the PSP scope, providing input on the survey design and dissemination strategy, overseeing the categorization and organization of submitted questions, and supporting the final prioritization process. Members of the steering group were selected through a purposeful targeting of researchers, patient and public representatives, healthcare professionals, researchers, policy makers, and key organizations in women+ health research from across the Maritime Provinces.

*Step 2: Gathering Uncertainties*

To collect uncertainties, data will be gathered through a region-wide open-access online survey available to individuals residing in Nova Scotia, New Brunswick, and Prince Edward Island. All participants will review and provide informed consent electronically prior to participating in the study. Consistent with established PSP methodology, no predefined sample size has been set; rather recruitment will focus on obtaining diverse perspectives across interest holder groups and geographic regions [23]. A pragmatic approach will be used, with survey recruitment focused on maximizing inclusivity across groups and provinces over six weeks. Participants may choose to provide their email address if they wish to receive updates about the project or be involved in later stages of the PSP, including the interim ranking survey and/or final consensus workshop. Participant email addresses will not be linked to survey responses. Immediately following consent, participants will be directed to the online survey.

The survey has two components. First, the survey will collect demographic information to monitor representation across populations and interest holder groups. These data will be used descriptively to assess and describe the diversity of respondents and guide targeted recruitment efforts, rather than to make subgroup comparisons or draw conclusions about specific populations. Demographic items will include age, sex, gender identity, sexual orientation, race, education level, and province of residence. Participants will also be asked to self-identify their respondent group(s), which include a person with a lived experience as a woman+, a caregiver of a woman+, a friend or family member of a women+, a healthcare professional, a researcher, a policy maker, and/or a member of the public.

Second, the survey will solicit unanswered research questions related to women+ health in the Maritime provinces by asking participants to identify what questions about women+ health in the Maritimes they believe should be prioritized for future research. Participants will be informed that we are seeking questions related to all aspects of women+ health in the Maritimes, including physical, mental, emotional, social, and cultural factors. Participants may submit up to ten responses at a time and may complete the survey more than once for the submission of additional research questions. During data processing, identical or highly similar duplicate submissions will be identified and removed to minimize overrepresentation of repeated questions and ensure data quality. The survey will provide examples of women+ health questions that focus on broader research priorities, including questions on physical, mental, emotional, social, or cultural health factors.

The survey will be administered online using REDCap (Version 15.5.36) [24] and will be available in both of Canada’s official languages (English and French). While this approach supports broad accessibility, it may limit participation from individuals who speak other languages, including some newcomer populations. To help mitigate this, targeted recruitment will be conducted through community organizations serving diverse and newcomer populations, which may support awareness of the study through trusted networks and assist individuals in accessing and understanding the survey. While the survey is only available in English and French, these strategies aim to enhance engagement among individuals who may otherwise face barriers to participation.

Eligibility criteria and recruitment strategies are informed by equity, diversity, inclusion, and accessibility (EDIA) guidance and sex- and gender-based frameworks to promote diversity and inclusivity across age, gender identity, geography (urban and rural), province, cultural background, and lived experience, with the aim of ensuring broad representation of perspectives relevant to women+ health across the Maritime provinces [25,26,27]. Recruitment material uses inclusive language to encourage participation from women+ and gender-diverse individuals, and targeted outreach will be conducted through community organizations and health networks serving diverse populations.

To support participation from historically underrepresented populations, outreach will also be conducted through organizations and community groups serving diverse populations across Nova Scotia, New Brunswick, and Prince Edward Island. Recruitment strategies will aim to engage these groups through targeted dissemination of the survey via healthcare networks, research institutions, community organizations, and advocacy groups across the Maritime provinces. This may include promotion through: (1) partner organizations and advisory networks (e.g., the Maritime SPOR SUPPORT Unit); (2) community-based organizations and health authorities across the Maritime provinces (e.g., Perinatal NB, IWK Health); (3) social media platforms (e.g., Meta); and (4) email outreach and newsletters. A curated list of regional organizations serving diverse and underrepresented populations (e.g., racialized, immigrant, low-income, and gender-diverse populations) has been developed to guide outreach and dissemination of the survey to promote participation from a range of perspectives. Efforts will also be made to reach women+ who do not have easy online access through dissemination via community and health organizations. Recruitment materials will emphasize that the survey can be completed at any time to support participation from women+ who may be postpartum, caregiving for young children, or experiencing health-related fatigue.

The study team acknowledges that, in the absence of formal partnerships with specific communities, the survey may not fully capture the priorities of all groups. Findings will be interpreted with attention given to principles of equity, inclusion, and data sovereignty. Survey responses will be monitored throughout the recruitment period to assess representation across respondent groups and inform additional outreach efforts if certain perspectives are underrepresented. Representation will be assessed using key demographic indicators (e.g., gender identity, racial background, sexual orientation, province, and participant role), and recruitment strategies will be iteratively adapted to enhance diversity across these groups where needed. Adequacy of diversity will be determined based on achieving broad representation across these indicators.

To help ensure data quality and reduce the likelihood of fraudulent or automated responses, the survey will incorporate security and verification measures (e.g., CAPTCHA v3 and attention checks). Responses will also be reviewed during data cleaning for indicators of potential duplicate or invalid entries.

*Step 3: Data process & verifying uncertainties*

Following completion of the initial survey, all submitted questions will undergo a structured data cleaning, review, and refinement process conducted by the research team. Survey responses will be cleaned to ensure accuracy and consistency, and submitted questions will be reviewed to remove duplicate, overlapping, or out-of-scope submissions. Duplicate submissions will be identified based on identical or highly similar wording and removed. Where appropriate, similar or overlapping questions will be grouped and consolidated into a single researchable uncertainty where they reflect a shared underlying concept.

If needed, refined questions will be rewritten in plain-language format to ensure clarity while preserving the original intent of participant submissions. Submissions will be excluded if they fall outside the defined scope of women+ health, do not represent a researchable question, or are insufficiently clear to interpret, with reasons for exclusion documented to support transparency and reproducibility. All in-scope uncertainties will be retained regardless of the frequency with which they are submitted. This approach ensures that less commonly reported or less visible health concerns are not excluded and are given equal consideration during subsequent prioritization stages. To maintain consistency and minimize bias, two members of the team will review all submissions independently, resolving differences through discussion or by involving the Steering Group when necessary.

The refined list of questions will be reviewed against existing literature, including randomized controlled trials, systematic reviews, and/or clinical practice guidelines in PubMed to determine whether uncertainties have already been addressed by current research, both within the Maritimes and beyond. This process will focus on whether the specific uncertainty has been answered by existing research evidence, rather than whether a broader topic area has been studied. Questions identified as clearly addressed by existing research evidence will be removed, while those that remain partially or wholly unanswered will be retained. Determinations will be made through review by the research team and discussed with the Steering Group to ensure consistency and transparency. This process will result in a consolidated “long list” of verified research uncertainties related to women+ health in the Maritimes. Any research questions removed at this stage will be reported separately and justification of evidence will be noted for transparency.

*Step 4: Interim priority setting*

An interim prioritization survey will then be developed and disseminated using the same online platform (REDCap [24]) and recruitment strategies as the initial survey. Participants will be asked to review the long list of uncertainties and to rank or select the questions they believe are most important for future research. Uncertainties will be grouped by women+ health theme (e.g., cancer, perinatal, heart health) under which questions will be listed, and participants will be able to select the themes that they want to vote on.

Participants will rank the importance of each question within themes using a 5-point Likert scale from 1 (“not important”) to 5 (“very important”). At the end of this step, Likert-scale responses will be summarized using descriptive statistics (e.g., mean scores and frequency distributions), and uncertainties will be ranked based on aggregated scores to identify the highest-priority items within each theme.

The highest-ranked uncertainties will be used to generate a shortlist of approximately 20–25 research priorities per thematic area. These shortlisted uncertainties will then be compiled into a consolidated list to be considered during the final priority-setting workshop.

*Step 5: Final priority setting workshop*

The final phase will involve a facilitated one-day consensus workshop conducted using a hybrid format (in-person with a virtual participation option). Approximately 30 participants will be purposively selected to reflect diversity across interest holder roles (women+, caregivers, healthcare professionals, researchers, policymakers, and members of the public), as well as variation in gender identity, age, geography, and lived experience related to women+ health. We will also offer a virtual option for those who cannot travel to Halifax, Nova Scotia or if interest exceeds 30 individuals.

Workshop participants will be provided with the shortlisted research questions in advance of the meeting to allow for reflection and preparation. The workshop will be facilitated using participatory methods (e.g., journey mapping, dot-voting, empathy mapping) using a combination of small group work and full-team sessions to support diverse contributions for participants in-person and online [28]. These approaches are commonly used in participatory research and PSPs to support inclusive dialogue and shared decision-making [29].

To mitigate potential power imbalances between participant groups (e.g., individuals with lived experience, healthcare professionals, and researchers), structured facilitation strategies will be used, including role-balanced small group discussions, guided dialogue, and iterative ranking exercises that allow participants to independently reflect and contribute prior to group discussion. These approaches are intended to support equitable participation and minimize the influence of hierarchical dynamics during the consensus process. The workshop will be facilitated by an individual with experience in equity-focused and trauma-informed facilitation to support inclusive, respectful, and psychologically safe engagement among participants.

Through iterative discussion and ranking, participants will collaboratively review the consolidated shortlist of uncertainties and work toward reaching consensus on a final, ranked Top 10 list of research priorities for women+ health in the Maritime provinces. While the goal of this process is to achieve consensus, differing perspectives and areas of disagreement will be documented throughout the workshop to ensure that a range of viewpoints is captured and considered in the interpretation of results. The outcome of this phase will be a set of community-endorsed research priorities that reflect shared decision-making and can be used to guide future research, funding, and policy initiatives in the region.

### 2.4. Data Management

All survey data will be collected and managed using REDCap (Research Electronic Data Capture; Vanderbilt University [24]), a secure, web-based data capture platform approved by the host institution. Data will be stored on secure institutional servers, with access restricted to authorized members of the research team. Any identifying information (e.g., contact details provided for future engagement) will be stored separately from survey responses and will not be linked to individual submissions during analysis.

Quantitative demographic data will be analyzed using descriptive statistics to summarize participant characteristics and assess representation across interest-holder groups and provinces. Descriptive summaries of responses may be used to assess representation across participant groups; however, no formal subgroup analyses will be conducted, as the aim of this PSP is to identify shared priorities across interest-holder groups rather than to compare differences between groups. Responses from the interim prioritization survey will be examined descriptively at the overall sample level to identify general patterns in priority rankings. Statistical analyses will be conducted using IBM SPSS Statistics version 21.

All analytic procedures, including criteria for determining in-scope and out-of-scope submissions and approaches to consolidating uncertainties, will be finalized prior to data analysis and documented to support transparency, reproducibility, and auditability. All findings will be reported according to the reporting guideline for priority setting of health research (REPRISE) guideline [30]. Any protocol amendments related to analysis will be reported.

## 3. Expected Outcomes and Impact

This study aims to produce a community driven, regionally relevant list of the top research priorities for women+ health in the Maritime provinces that reflect the needs and lived experiences of women+ in the Maritime provinces. The identified priorities are intended to inform future funding applications, policy discussions, and program planning within these networks. Recognizing that health system needs and research landscapes evolve over time, the identified priorities may require future review and updating. This study provides a foundation for ongoing priority-setting efforts and may inform future iterations or follow-up initiatives as new evidence and contextual factors emerge.

The findings are expected to have several potential impacts, including, but not limited to, informing the development of future research agendas by highlighting high-priority questions that remain unanswered in women+ health and helping to ensure that studies address areas of greatest need and relevance. Further, this study may provide actionable insights for health system planners, policymakers, and healthcare organizations to align services and programs with community-identified priorities. This study may also contribute to strengthening partnerships and networks among women+, researchers, healthcare professionals, and decision makers, fostering ongoing collaboration and patient-oriented research. While this PSP is not setting out to identify unique differences in priorities across groups, the study will aim to ensure that the resulting priorities reflect a broad range of perspectives and lived experiences related to women+ health in the Maritime provinces. Collectively, the outputs of this study are intended to inform a strategic foundation to guide future research, funding, and policy decisions, while demonstrating a replicable and inclusive approach to priority settings that could be adapted in other regions or health contexts.

Beyond generating the priority list for academic publication, the project will actively mobilize these findings through multiple pathways. Results will be shared with study participants and community partners through accessible lay summaries, infographics, and project updates distributed via the project website and partner organizations and networks. The findings will also be embedded in future grant applications and research programs led by academic and community partners and disseminated through targeted policy briefs and presentations to inform decision-makers within Nova Scotia Health, Horizon/Vitalité, Health PEI, and IWK Health. These activities aim to ensure that interest holders who contribute to the priority-setting process remain informed about how their input shaped the final priorities and how the results may influence future research and health system planning.

Despite the potential for positive outcomes, there are some limitations to acknowledge. First, there is a potential risk of “priority fatigue,” where repeated consultation processes may reduce participant engagement over time. To mitigate this, efforts will be made to sustain engagement beyond the initial priority-setting process through ongoing communication with participants, integration of findings into active research programs, and continued collaboration with community and health system partners. These strategies aim to demonstrate how participant contributions are translated into action and to maintain trust and engagement over time. Another limitation is the demographic information collected is quite brief to maximize survey completion. While additional factors such as relationship status and social support may influence health experiences and access to care, these were not included in the demographics but nevertheless, may be important to consider in future research.

## 4. Conclusions

This protocol presents a systematic, transparent, and inclusive approach to identifying and prioritizing research questions in women+ health across the Maritime provinces of Canada. The resulting Top 10 research priorities are expected to: (1) Inform evidence-based research funding decisions; (2) Guide collaborative, patient-oriented research initiatives; (3) Support the development of equitable, regionally relevant women+ health research agendas; and (4) Strengthen networks and capacity for ongoing interest holder engagement in women+ health research. By centering diverse voices and systematically translating community-identified needs into actionable priorities, this study provides a replicable model for inclusive priority setting that can be adapted to other populations, regions, or areas of health research.

## Data Availability

This protocol paper does not report any data. Data sharing is not applicable to this article.

## Abbreviations

The following abbreviations are used in this manuscript:

PSP	Priority Setting Partnership
JLA	James Lind Alliance
